# Human Being Detection from UWB NLOS Signals: Accuracy and Generality of Advanced Machine Learning Models

**DOI:** 10.3390/s22041656

**Published:** 2022-02-20

**Authors:** Gianluca Moro, Federico Di Luca, Davide Dardari, Giacomo Frisoni

**Affiliations:** 1 Department of Computer Science and Engineering (DISI), University of Bologna, 47521 Cesena, Italy; giacomo.frisoni@unibo.it; 2 Department of Electrical, Electronic, and Information Engineering (DEI), University of Bologna, 47521 Cesena, Italy; federico.diluca@studio.unibo.it (F.D.L.); davide.dardari@unibo.it (D.D.)

**Keywords:** ultra-wideband (UWB), non-line-of-sight (NLOS), machine learning, transfer learning, human detection

## Abstract

This paper studies the problem of detecting human beings in non-line-of-sight (NLOS) conditions using an ultra-wideband radar. We perform an extensive measurement campaign in realistic environments, considering different body orientations, the obstacles’ materials, and radar–obstacle distances. We examine two main scenarios according to the radar position: (i) placed on top of a mobile cart; (ii) handheld at different heights. We empirically analyze and compare several input representations and machine learning (ML) methods—supervised and unsupervised, symbolic and non-symbolic—according to both their accuracy in detecting NLOS human beings and their adaptability to unseen cases. Our study proves the effectiveness and flexibility of modern ML techniques, avoiding environment-specific configurations and benefiting from knowledge transference. Unlike traditional TLC approaches, ML allows for generalization, overcoming limits due to unknown or only partially known observation models and insufficient labeled data, which usually occur in emergencies or in the presence of time/cost constraints.

## 1. Introduction

Ultra-wideband (UWB) radars have found application in several fields over the last decade, such as the biomedical and industrial ones, thanks to technological progress. Specifically, the UWB-radar transmits exploratory signals with a much larger spectrum than conventional radars, resulting in significant advantages in localization owing to its centimeter-level range resolution, multipath robustness, high penetration ability, low cost and energy consumption. One of the most impressive characteristics of these systems is their ability to locate human beings by detecting small chest movements while breathing [1], even in non-line-of-sight (NLOS) conditions. This capacity can be helpful in many situations. Three examples are recognizing moving human targets in a surveyed area, remotely monitoring patients’ vital functions, and identifying survivors that are trapped under rubble after an earthquake or a catastrophe.

Classical model-based TLC approaches for human detection usually rely on scenario-specific studies and optimizations, justifying the need for new and easily generalizable data-driven solutions. This paper presents an extensive measurement campaign with different environments, obstruction materials, and body orientations using an UWB-radar. We examine various machine learning (ML) and deep learning (DL) methods to find the best classifier that can distinguish between the presence and absence of a normally-breathing human being under NLOS radio propagation conditions by adequately processing the backscattered UWB signal. We assess model performance with typical classification metrics. Finally, the general validity of the best models presented in the paper is investigated by applying transfer learning techniques.

The remainder of the paper is structured as follows. In Section 2, we describe related work. In Section 3, the measurement campaign set-up is illustrated. The collected data, the validation methods, and the main evaluated ML algorithms are introduced in Section 4, whereas in Section 5, NLOS human being classifiers are derived, examined, and compared with a standard periodogram-based TLC algorithm. Section 6 discusses the obtained results and the advantages of the proposed models. In Section 7, conclusions are drawn.

## 2. Related Work

The problem of human being detection in NLOS settings has been studied in several works. The first literature mention can be found in [2], where Ossberger et al. proposed a method for vital parameter detection through a UWB pulse radar, using the Continuous Wavelet Transform (CWT) with special background subtraction. This technique allowed for respiration detection up to a distance of 5 m, including behind walls.

In [3], Yarovoy et al. conducted an in-depth investigation of the scattering characteristics of the human body. They implemented a novel motion/breathing detector based on spatial variation measurements. This contribution did not require separating the signal component backscattered by the body from that scattered by the environment (clutter). It worked reliably in an environment characterized by multipath propagation. The two variables used to locate a human being were the direction and time of arrival.

In [4], Zaikov et al. proved that it is possible to detect trapped persons under rubble by means of an M-sequence UWB-radar, inlucuding in wet debris. Specifically, the authors detected a breathing person by evaluating the radargram variations after a signal-enhancing step.

After the success of UWB-radar technology for through-wall detection, in [5], Li et al. proposed a method based on Fast Fourier Transform (FFT). In this paper, the authors showed how to extract the central frequencies of life signals and locate the position of human targets with high accuracy.

In [6], Schleicher et al. introduced three methods of movement determination. Among these, the slope-to-slope tracking between direct coupling and object reflection was found to be the most suitable (i.e., slightest detection error).

In [7], Rittiplang et al. investigated a through-wall S-band 1-Tx/5-Rx UWB switched-antenna-array radar scheme for detecting stationary human subjects from respiration. The signals were preprocessed to remove unwanted noises, and statistical variance analysis was undertaken for 3D–2D array conversion. A back-projection algorithm was implemented to reconstruct the images, refined by the Sinc filter. The results revealed that the through-wall UWB switched-antenna-array radar could distinguish between human subjects and mannequins.

Another method that involves the spectrogram analysis to detect and localize human targets in NLOS condition was explained in [8]. In this paper, the Curvelet Transform (CT) was applied to remove the direct wave and the background clutter. Subsequently, Singular Value Decomposition (SVD) was used to reduce the noise in the weak life signal. Finally, a spectral analysis with FFT was carried out to separate and extract the breathing and heartbeat frequency.

In [9], Casadei et al. proposed a spectrogram approach to facilitate the detection of a breathing person and their estimated distance from the radar. In other words, they showed breath activity detection rates with the corresponding threshold values by using a periodogram. Performance was evaluated in a realistic NLOS scenario in terms of detection accuracy and false alarm rate, achieving a score of 70% and 5% for brick walls, respectively. Another classic method is shown in [10], in which the authors reported a minimum missed rate equal to 2%.

Classical signal detectors [11,12,13] have also proven effective in radars, sonars, and communications. Per contra, simple radio signal measures such as Received Signal Strength Indicator (RSSI) readings are heavily affected by obstruction at the LOS between a transmitter and receiver [14], making them unsuitable under the analyzed conditions. In this sense, Barral et al. [15] confirmed the significant degradation effect caused by NLOS conditions in UWB-based distance estimation.

A comprehensive review of the latest research on a UWB through-wall radar has been presented by Yang et al. [16], covering human detection theory and signal-processing algorithms such as human vital sign measurement. For human detection (i.e., determining the absence or presence of a target in the inspected radar signals) with NLOS sensing, the authors identified three main strategies: (i) constant false alarm rate (CFAR), where the goal is determining the power threshold above which any return can be considered to likely originate from a target as opposed to one of the spurious sources; (ii) statistical characteristics of the received signal (skewness, kurtosis, energy, etc., also used in this work); (iii) multipath models of the radar return signal.

Even though the results achieved by the above-mentioned papers are quite satisfactory in the dissected scenarios, we would like to highlight the need for a suitable backscatter signal model and an empirical tuning of its parameters. Unfortunately, the model and the tuning process are scenario-specific. Hence, such classic methods—which usually imply the use of an analytical model to prepare the signal for a threshold breath activity detector—might fail under conditions that are different from those designed for them.

With the aim of pursuing a better generalization capacity, the other category concerns machine learning approaches. ML techniques can effectively deal with different NLOS conditions caused by distinct environments, wall materials, and target orientations, recognizing patterns that are difficult to find otherwise.

One of the very first approaches to adopt ML and WiFi Sensing to classify and estimate human respiration activity dates back to 2017 [17]. In this paper, Khan et al. developed a Convolutional Neural Network (CNN), reaching an accuracy of 94.85%. However, they considered only line-of-sight (LOS) conditions and worked on a dataset of limited size.

In [18], the Autoencoder NN architecture exhibited a maximum accuracy of 99%. Similarly, in [19], Ding et al. evaluated five supervised learning algorithms. Even in this case, the dataset was small, and an Autoencoder was proved to be the best method, reaching 99% accuracy.

Park et al. [20] proposed a transfer-learning-based UWB NLOS identification scheme for unmeasured environments, showing an accuracy similar to deep learning models trained with 30× data and 48× computation times. However, they analyzed only multilayer perceptrons and convolutional neural networks, without exploring additional architectures and human detection tasks.

The main limitation of the ML contributions in the literature is the dataset dimension, which is typically relatively small and not suitable for AI techniques, which are are notoriously data-hungry. Unlike previous works, we aim to obtain a larger dataset from an extensive measurement campaign by considering multiple scenarios and settings. In addition, we analyze a broader spectrum of ML and DL methods, evaluating their generalization properties through transfer learning techniques.

## 3. Measurement Campaign Setup

We performed an extensive measurement campaign in different scenarios and target displacements to create a comprehensive database for applying and evaluating deep learning algorithms as human being classifiers.

**Figure 1:**
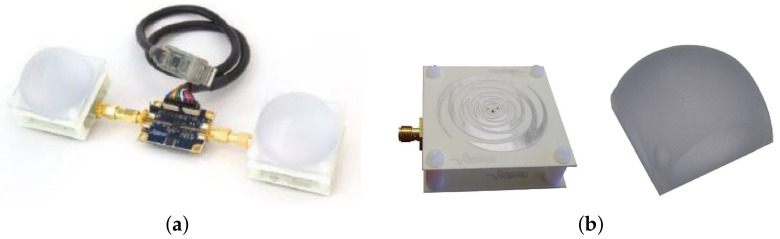
UWB-radar instrumentation. (**a**) Radar Novelda NVA-R661. (**b**) Sinuous antennas and dielectric lenses.

We employed the Novelda NVA-R661 radar development kit—based on NVA-6201 chip and operating in the 6–8.5 GHz band—as the commercial device for the campaign (Figure 1). It comprehends an IO module for PC communication and a software library. In addition, the kit provides a couple of sinuous antennas with dielectric lenses for narrowing the radiation pattern. Antenna and transceiver datasheets are available at https://www.laonuri.com/en/product/sinuous/ and https://www.laonuri.com/en/product/nva-r661/, accessed on 22 December 2021.

Notice that the chosen bandwidth is 2.5 GHz and, by definition, is UWB. In fact, according to FCC, a signal is UWB if its bandwidth is larger than 500 MHz. Unfortunately, international regulations do not allow for the exploitation of the whole 3–10 GHz band, whose usage is restricted to some sub-bands depending on the area and with different limitations (e.g., detection-and-avoidance (DDA) or low-duty cycle (LDC) schemes). Moreover, the available chipsets and standards allow for a maximum bandwidth of about 1.6 GHz (however, this is typically 500 MHz). Therefore, in our opinion, the choice of the 6–8.5 GHz is reasonable because (i) it is allowed worldwide and without DDA/LDC restrictions; (ii) it is also available at a practicable low cost today, thus making the results valid for affordable technology.

The selected radar is general-purpose and can be programmed and configured for sensing applications of up to 60 m with 4 mm spatial resolution. With equipped lenses, the typical antenna gain is 6.7 dBi. Furthermore, it is a bistatic radar; it includes transmitter circuits to generate a Gaussian impulse signal in the 6–8.5 GHz band, a receiver to collect the reflected (backscattered) signal, and special circuitry to gather the range data for target detection and imaging.

The Novelda Radar Software Bundle installation program contains all necessary instructions to interface with the radar and documentation. Moreover, the software library gives the developer easy access to the complete functionality of the Novelda Radar using RadarScope GUI application. This software allows all the control parameters for transmission to be managed, such as pulse generator frequency, spatial length, and Pulse Repetition Frequency (PRF). The radar periodically sends a large number of UWB pulses at frequency PRF. Then, it collects the signal samples backscattered by the surrounding environment in a limited observation window of duration $T_{f}$, starting from a selectable time offset. In our tests, we have chosen PRF = 6 MHz so that, according to the Radar datasheet, 69 waveforms/s can be acquired. The observation window duration is $T_{f}$ = 7.11 ns, corresponding to 1.06 m at the sampling time of $t_{s}$ ≈ 30 ps. The total number of samples collected per observed window is *K* = 256.

Experimental data were collected in different indoor scenarios in the main building of the School of Engineering at the University of Bologna, Cesena Campus. First of all, we considered a *static* measurement, which means that the radar was placed still on a mobile cart at about 130 cm from the ground. The target was always placed *d* = 20 cm far behind the obstacles, which was *r* = 30, 60 and 90 cm from the radar. Using *s* as the material thickness, we considered different obstacles. In particular, we chose to consider: double-glazing *s* = 10 cm, wooden door *s* = 3 and 5 cm, brick wall *s* = 15 cm, and glass window *s* = 2 cm. In the second scenario, we inspected a more realistic situation in which the radar was handheld at different heights, so that small movements made the acquisition *dynamic*. For this experiment, we ignored the double-glazing window.

**Figure 2:**
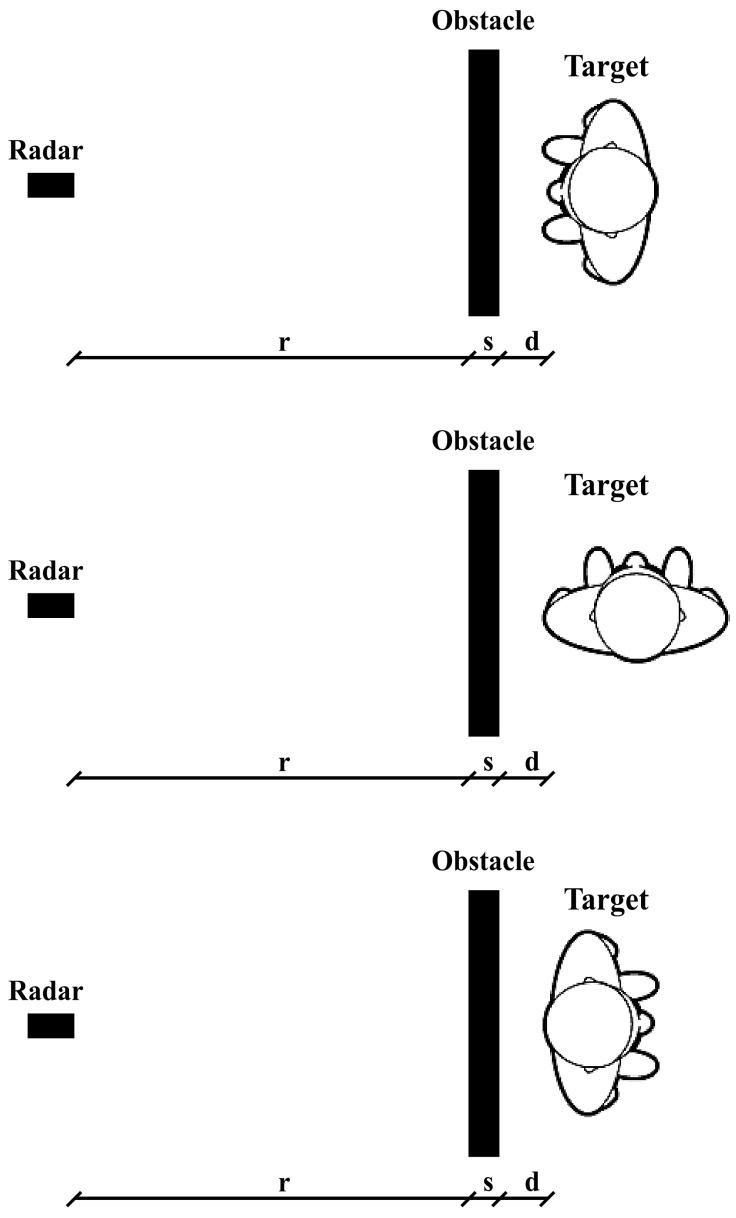
The different body orientations considered within our experiments for through-wall human detection. Note: r = radar-obstacle distance; s = obstacle thickness; d = target-obstacle distance.

As depicted in Figure 2, various measurements were taken to consider different body orientations. For each combination of obstacle type, target orientation, and radar distance, *N* = 512 consecutive waveforms were collected for a total measurement time of ≈7 s. The selected measurement time was chosen as it is large enough to contain one breath in normal conditions. Three different body orientations were collected: (i) the person with his chest facing the radar; (ii) the person rotated 90 degrees; (iii) the person with their back to the radar. During the acquisitions, the target people were asked to vary their breathing frequency.

In Table 1, we show the measurement campaign setup. We obtained two datasets in the form of I×J matrix *M*. For the *static* case, the number of total instances is *I* = 23.552; for the *dynamic* one, *I* = 17.408. *J* = 256 (*K*) for both cases. The number of rows *I* is obtained by concatenating every pulse set from the measurement campaign, discarding the erroneous ones (i.e., absence of information content and high presence of noise) based on a qualitative inspection.

### 3.1. Data Processing and Datasets Creation

**Figure 3:**
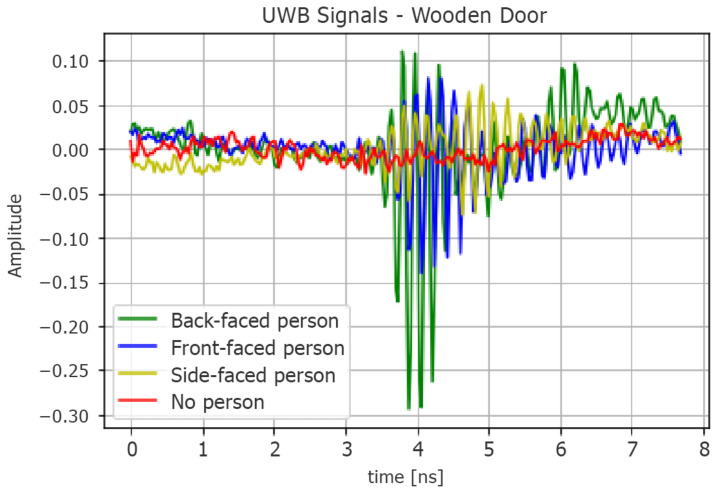
Example of time-domain pulses acquired in static condition.

Having arranged the labeled raw data in two matrices, we extracted three more datasets for each one of them. The starting matrices, which are 23,552 × 256, for the static case and 17,408 × 256 for the dynamic one, will be referred to as the datasets containing the signals in the time domain (raw data). An example of such pulses is shown in Figure 3, in which we reported four signals that were acquired in the *s* = 3 cm wooden door scenario and static condition showing the normalized amplitude on the observation window $T_{f}$.

**Figure 4:**
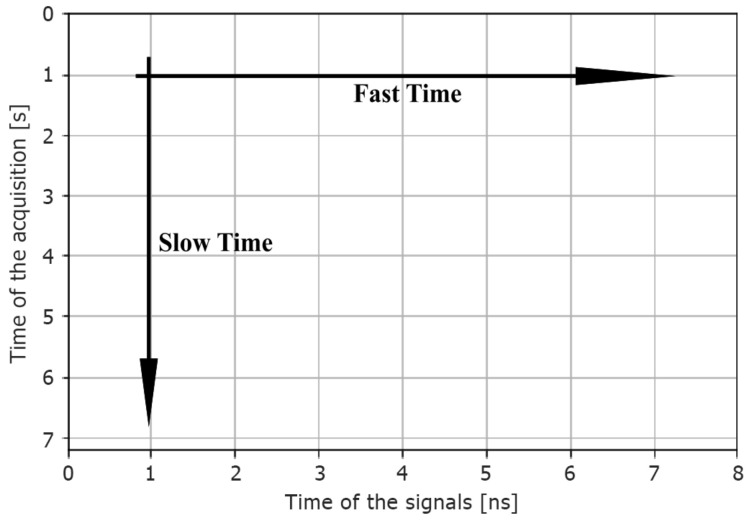
Slow Time and Fast Time.

A second and a third dataset were obtained from the raw data by computing the horizontal and vertical *Fast Fourier Transform* (FFT). Particularly, the horizontal FFT was calculated through the *Fast Time* direction, which means over the time of each interrogation signal. The vertical FFT was applied over the *Slow Time* direction, which indicates the time of pulse acquisition. Specifically, the horizontal FFT should capture the changes in the signal spectrum due to obstacle/person. In contrast, the vertical FFT should capture the slow variation in the backscatter signal caused by breath or movements (Doppler spectrum). In Figure 4, we clarify this concept.

To create the fourth dataset, we extracted a set of statistical attributes from the waveforms, which are distinctive for UWB signals analysis, as shown in [21]. This elaboration is also useful when obtaining new, possibly significant data from the raw signals. In Table 2, we present the statistics we used as attributes. Below, we list their definitions.

*Skewness* is a measure of the lack of symmetry in a dataset. A dataset, or distribution, is symmetric if it looks the same to the left and right of the center point. *Kurtosis* is a measure of whether the data are peaked or flat relative to a normal distribution. In particular, datasets with high kurtosis tend to have a distinct peak near the mean, then decline rather rapidly, followed by heavy tails. Datasets with low kurtosis are more likely to have a flat top near the mean rather than a sharp peak. Taking $x_{i}$ as the signal samples, $\bar{x}$ as the mean, $\sigma_{X}$ as the standard deviation and *n* as the number of datapoints, skewness $b_{1}$ and kurtosis $g_{2}$ are defined as follows:

(1) $$b_{1}=\frac{\sum_{i=1}^{n}{\left(x_{i}-\bar{x}\right)}^{3}}{\left(n-1\right)\sigma_{X}^{3}}$$

(2) $$g_{2}=\frac{\sum_{i=1}^{n}{\left(x_{i}-\bar{x}\right)}^{4}}{\left(n-1\right)\sigma_{X}^{4}}$$

whereas the *energy* of the signal is:

(3) $$E=\sum_{i=1}^{n}x_{i}^{2}$$

Considering the above, we obtained four datasets for both cases, containing: raw data (time-domain waveforms), horizontal FFT signals (horizontal–frequency domain), vertical FFT signals (vertical-frequency domain or Doppler spectrum), and attributes extracted from the raw data (statistic domain).

The obtained measurement data are available for public use: https://github.com/disi-unibo-nlu/uwb-nlos-human-detection, accessed on 22 December 2021.

## 4. Materials and Methods

Deep learning is part of a broader family of machine learning methods based on artificial neural networks. Learning can be supervised, semi-supervised, or unsupervised. Before applying algorithms to discover patterns, the target dataset must be large enough. In the preprocessing phase, the target dataset is created and reduced into *feature vectors*, one for each observation. Then, a set of such vectors, also called *instances*, can be fed to a learning algorithm, which treats them as examples from which to extract the underlying patterns. Many learning algorithms exist, based on different theoretical bases and yielding to models of distinct formats.

We consider two different learning approaches according to the nature of the input data. *Supervised learning* algorithms take labeled instances as input. Therefore, each instance must be labeled with the class of interest. In our case, waveform instances are marked as either “Person YES” or “Person NO” to allow the final model to classify subsequent preprocessed waveforms into one of these cases. *Unsupervised learning* algorithms take unlabeled instances as input, with no additional information, and divide the given data into *clusters*. In a general vein, they are employed in a wide range of AI-based applications, like topic detection [22] and job recommendation [23]. These algorithms maximize the similarity between instances of the same cluster and minimize that between instances of different clusters. As a result, each cluster will contain instances with specific, prominent characteristics. The advantage of this type of learning is that it can provide a high-level classification, avoiding the labeling phase, which is usually time-consuming and made by users. By contra, a disadvantage is that the unsupervised approach yields a more generic model that subdivides instances into groups with no predefined meaning. The collected labels are used only to calculate unsupervised algorithm evaluation metrics. These models are expected to be far less accurate than the supervised ones, but not less interesting, due to their ease of training. In fact, the considerable advantage of using clustering methods is that they do not require training data to be labeled. We implemented each model in Keras.

In the following, we first describe how we preprocessed the raw data to create different datasets; then, we present the specific supervised and unsupervised learning methods that we examined.

### 4.1. Validation Methods

To evaluate how our classification models make predictions, we considered the Confusion Matrix (CF) and the associated metrics. A confusion matrix is a summary of prediction results on a classification problem. The number of correct and incorrect predictions are summarized with count values and divided by each class. We can formally define the CF-associated metrics as TP, TN, FP, and FN, which are the well-known True Positives, True Negatives, False Positives, and False Negatives, respectively. In this case study, the presence of a human being behind the obstacle will be considered a Positive, while the absence of the subject will be a Negative. The first and main metric is the *Accuracy*, which is defined as follows:

(4) $$Accuracy=\frac{TP+TN}{TP+FP+TN+FN}$$

The problem with accuracy is that it assumes equal costs for both kinds of errors. For this reason, we also considered Precision and Recall.

(5) $$Precision=\frac{TP}{TP+FP}$$

(6) $$Recall=\frac{TP}{TP+FN}$$

High Precision indicates that an example labeled as positive is indeed positive (small FP). High Recall indicates that the class is correctly recognized (small FN). F1 Measure is a measure that represents both Precision and Recall.

(7) $$F1=2\cdot \frac{Precision\cdot Recall}{Precision+Recall}$$

This is usually nearer to the smallest value. High recall and low precision mean that most positive examples are correctly recognized (low FN), but there are a lot of false positives. In contrast, low recall and high precision show that we miss a lot of positive examples (high FN), but those we predict as positive are indeed positive (low FP).

From the above metrics, it is possible to derive the *Receiver Operating Characteristic* (ROC curve) and the *Precision–Recall curve* (PRC). The former is a performance measure defined at various thresholds settings. This shows how capable a model is of distinguishing the classes. The higher the *Area Under The Curve* (AUC), the better the model is as a classifier. A PRC plots Precision against Recall and does not consider TN values.

The first validation method we applied to evaluate our models’ performance is the *Hold-Out Validation*. With Hold-out, the dataset is split into a train and a test set. The training contains the model’s data, whereas the test set is used to see how well that model performs on unseen data. The split we adopted is 70% of data for training and the remaining 30% for testing. Due to its time efficiency, we used this evaluation method for Neural Networks (NN) models evaluation.

Another more robust validation method we applied to evaluate our models’ performances is *K-Fold Cross Validation* (K-Fold CV). With K-Fold CV, a given dataset is initially divided into *K* subsets (folds). Each fold is then used as a test set, whereas the remaining ones are merged as the training set. This process is repeated until each fold has been used as the testing one. The results are *K* metrics: one for each analysis. Calculating the average of these parameters, we obtained a more solid measure of the model performances. In this work, we chose *K* = 5.

The subsequent chapters briefly describe the ML and DL algorithms that we evaluated as classifiers.

### 4.2. Gradient Boosting Decision Trees

A *Decision Tree* is a flowchart-like structure in which each internal node represents a “test” on an attribute, each branch represents the outcome of the test, and each leaf node represents a class label (decision). The paths from the root to the leaf represent classification rules. Tree-based learning algorithms are considered one of the best and most used supervised learning methods. Tree-based methods empower predictive models with high accuracy, stability, and ease of interpretation. Moreover, unlike linear models, they map non-linear relationships quite well.

*Gradient Boosting* is a ML technique for regression and classification problems, which produces a prediction model in the form of an ensemble of weak prediction models, which are typically decision trees. It builds the final model in a stage-wise fashion as other boosting methods do, and it generalizes them by allowing optimization of an arbitrary differentiable loss function. Decision Trees are very flexible and efficient algorithms, but a single Tree could easily be subjected to overfitting, making it difficult to generalize.

*Gradient Boosting Decision Trees* (GBDT) combine multiple Decision Trees predictions to generalize as good as possible the result. GBDTs are iteratively trained, one by one. The critical thing to notice is that, for the Decision Tree definition, searching for the best splits requires the generation and the evaluation of every possible split. Therefore, an analytical solution, which provides the best split without considering them all, does not exist. This computational issue is the most challenging aspect of the Decision Trees’ training.

There are several GBDT algorithms, which mainly differ from one another in the ways in which they avoid the evaluation of all the possible split. In this work, we considered three GBDT algorithms which proved to be excellent classifiers: *XGboost* [24], *LightGBM* [25], and *CatBoost* [26].

### 4.3. Feed-Forward, Recurrent, Autoencoder Neural Networks

A standard NN consists of many simple connected processors called neurons, each producing a sequence of real-valued activations. Input neurons are activated through sensors perceiving the environment, and others are activated through weighted connections from previously active neurons. Learning is about finding weights that make the NN exhibit the desired behavior. Depending on the problem and how the neurons are connected, such behavior may require long causal chains of computational layers, where each stage transforms (often in a non-linear way) the aggregate activation of the network [27]. *Feedforward Neural Network* (FNN) was the first and simplest type of artificial neural network conceived. In this NN, the information moves in only one direction, forward, from the input nodes, through the hidden nodes, and to the output nodes. There are no cycles or loops in the network.

**Figure 5:**
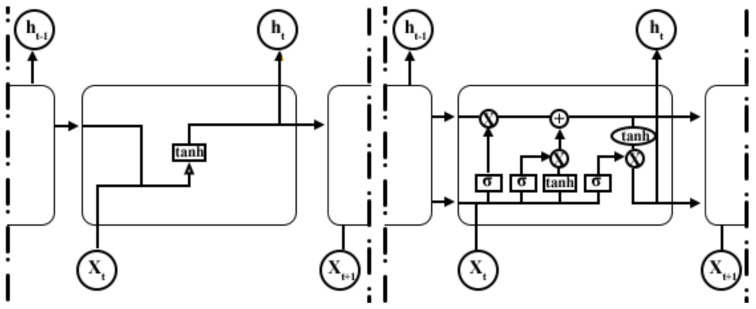
Difference between FNN (**left**) and LSTM Neurons (**right**).

*Long Short-Term Memory* (LSTM) is an artificial Recurrent Neural Network (RNN). Unlike FNN, LSTM has feedback connections [28]. A standard LSTM unit comprises a cell, an input gate, an output gate, and a forget gate. The cell remembers values over arbitrary time intervals, and the three gates regulate the flow of information into and out of the cell. LSTM networks are well-suited to classify processes and make predictions based on time series data since there can be lags of unknown duration between essential events in a time series [29,30]. We expect to obtain high-accuracy results on raw data with the LSTM model thanks to its ability to recognize temporal dynamic behavior in time-domain waveforms. The difference between FNN and LSTM neurons is shown in Figure 5.

**Figure 6:**
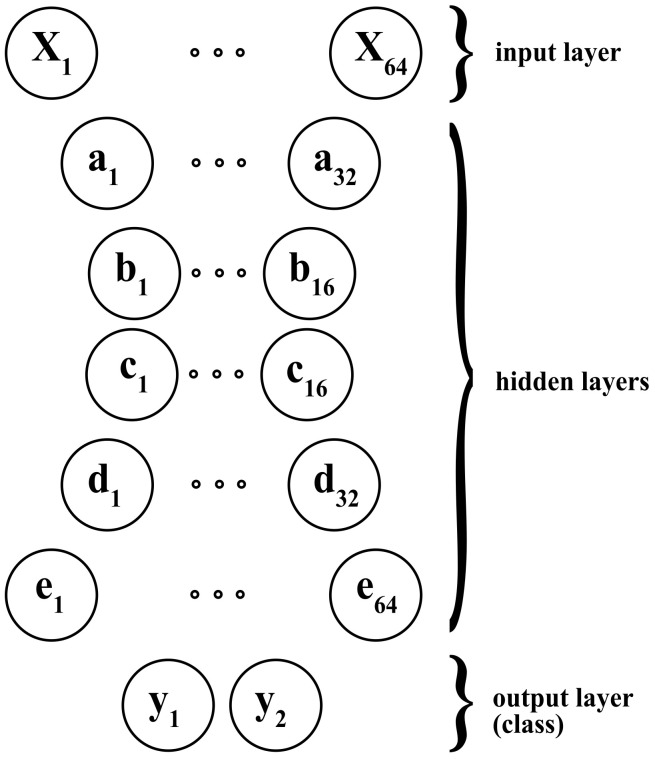
Implemented Autoencoder classifier architecture.

An *Autoencoder* (AE) is a type of NN used to learn efficient data codings in an unsupervised manner. An autoencoder aims to learn a representation (encoding) for a set of data, typically for dimensionality reduction, by training the network to ignore signal noise. Along with the reduction side, a reconstructing side is learned, where the Autoencoder tries to generate a representation that is as close as possible to its original input from the reduced encoding [31]. Several variants of the basic model exist to force the learned input representations to assume valuable properties. Autoencoders are effectively used to solve many applied problems, from face recognition to acquiring the semantic meaning of words. In this work, we used them to process time-series data, such as in [32]. In Figure 6 we depicted the Autoencoder NN architecture we developed as a classifier, which gives the input’s class as output. Notably, each letter corresponds to a layer, and the lowercase number stands for the associated neurons.

### 4.4. K-Means

*K-Means* (KM) is a simple unsupervised algorithm that is capable of clustering datasets very quickly and efficiently, often requiring only a few iterations. The primary input to the clustering algorithm is the number of clusters *K*. This parameter determines the clustering mechanism and how the clusters themselves are created. *K* is not always known a priori, but it is in this case study. We aim to let KM discover two clusters, one for each class of interest (“Person YES” and “Person NO”). Given a set of observations $x_{1},x_{2},\ldots ,x_{n}$, where each observation is a *d*-dimensional real vector, KM clustering partitions the *n* observations into K≤n sets *S* = $S_{1},S_{2},\ldots ,S_{K}$ to minimize the within-cluster sum of squares. Formally, the objective is to find:

(8) $$\underset{S}{\arg\min}\sum_{i=1}^{K}\sum_{x\in S_{i}}{∥x-\mu_{i}∥}^{2}=\underset{S}{\arg\min}\sum_{i=1}^{K}|S_{i}|VarS_{i}$$

where $\mu_{i}$ is the mean of points in $S_{i}$. This is equivalent to minimize the pairwise squared deviations of points in the same cluster:

(9) $$\underset{S}{\arg\min}\sum_{i=1}^{K}\frac{1}{2|S_{i}|}\sum_{x,y\in S_{i}}{∥x-y∥}^{2}$$

To equivalence between Equations (8) and (9) can be obtained from the identity:

(10) $$\sum_{x\in S_{i}}{∥x-\mu_{i}∥}^{2}=\sum_{x\neq y\in S_{i}}\left(x-\mu_{i}\right)\left(\mu_{i}-y\right)$$

Because the total variance is constant, this is equivalent to maximizing the sum of squared deviations between points in different clusters, which follows from the law of total variance [33].

### 4.5. Dimensionality Reduction Techniques: PCA and t-SNE

In statistics, machine learning, and information theory, *Dimensionality Reduction* is the process of reducing the number of random variables under consideration by obtaining a set of principal variables. For high-dimensional datasets, such as the ones we have, dimension reduction is usually performed before applying an unsupervised algorithm to avoid the effects of the *Curse of Dimensionality*. Dimension reduction can be achieved by using several techniques as a pre-processing step before applying the clustering approach. Dimensionality Reduction has several advantages. First of all, it reduces the time and storage space required. In the second instance, removing multi-collinearities improves the interpretation of the ML model parameters. Then, it allows for data visualization in lower-dimension domains, such as 2D or 3D.

In this work, we considered two Dimensionality Reduction techniques: *Principal Component Analysis* (PCA) and *t-distributed Stochastic Neighbor Embedding* (t-SNE).

*PCA* is a statistical procedure that uses an orthogonal transformation to convert a set of observations of possibly correlated variables into a set of values of linearly uncorrelated variables, called principal components. This transformation is defined so that the first principal component has the largest variance, and each succeeding component has the highest possible variance under the constraint that it is orthogonal to preceding components. Variance is directly proportional to the variability in the dataset. The resulting vectors are an uncorrelated orthogonal basis set. These vectors are linear combinations of the variables and contain *n* observations. An important note is that PCA is sensitive to the relative scaling of the original variables [33,34].

*t-SNE* is a nonlinear dimensionality reduction technique, well-suited to embedding high-dimensional data for visualization in a low-dimensional space of two or three dimensions. It models each high-dimensional object by a two- or three-dimensional point in such a way that nearby points refer to similar objects and distant points to dissimilar ones, with high probability. The t-SNE algorithm comprises two main stages. First, t-SNE constructs a probability distribution over pairs of high-dimensional objects so that similar objects have a high probability of being picked, whereas dissimilar points have a minimal probability of being selected. Second, t-SNE defines a similar probability distribution over the points in the low-dimensional map. It minimizes the Kullback–Leibler divergence between the two distributions with respect to the locations of the points in the map. Note that, while the original algorithm uses the Euclidean distance between objects as the base of its similarity metric, this could be changed as appropriate [35].

## 5. Experiments and Results

This section shows and compares the results we obtained by applying the above algorithms on each dataset in both static and dynamic cases. In the following tables, we present the Accuracy of each model we developed, together with Precision, Recall, and F1 values for both classes. Then, we highlight only the normalized CF and the ROC curve associated with our best result.

We considered eight datasets containing both signals in the time and frequency domains, as well as the attributes extracted from the waveforms. Moreover, we considered multiple subsets of features, which we chose among those described in Section 3.1. From now on, the two classes “Person YES” and “Person NO” will be shortened to “YES” and “NO”, respectively. In Table 3, data types and corresponding dataset names are shown.

### 5.1. Supervised Methods

**Figure 7:**
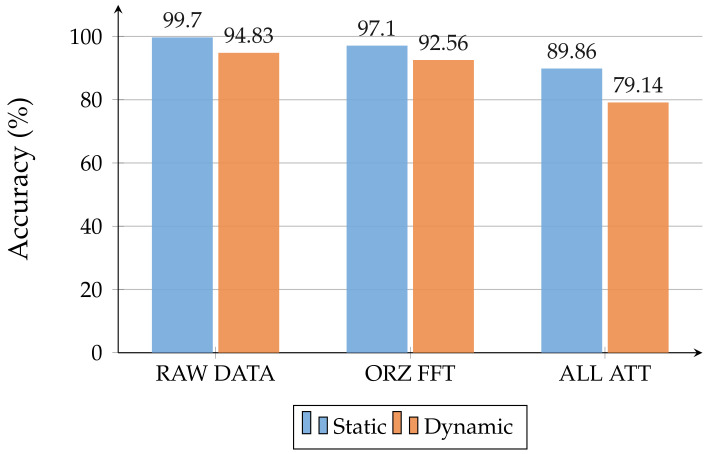
Comparison of best supervised models in static and dynamic cases.

**Figure 8:**
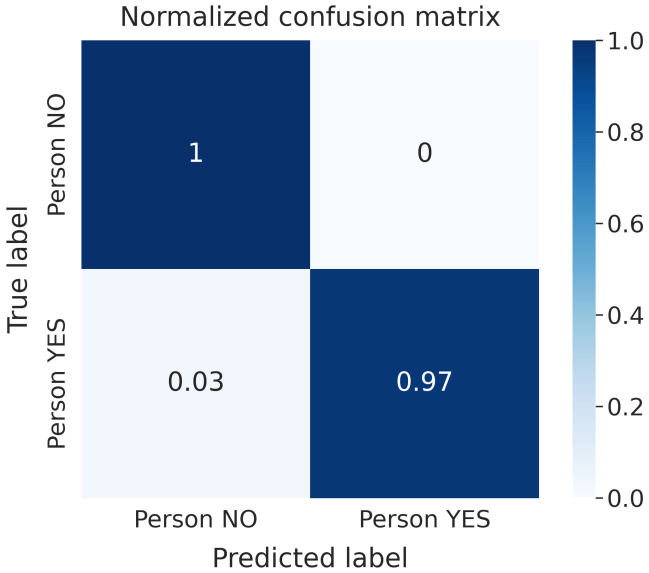
Normalized Confusion Matrix LSTM model—raw data, static case.

As expected, the comparison between Table 4 and Table 5 highlights that less accurate models are obtained when the radar is held by someone instead of being in a fixed position. In particular, a drop in accuracy of about 5% can be observed for NN architectures and 10% for GBDT models. This difference is shown in Figure 7. LSTM has proven to be the best NN working with raw data, thanks to its ability to recognize temporal dynamic behavior in time series data, e.g., time-domain waveforms. LSTM models achieved the best performance metric values among all the trained models. Figure 8 shows the normalized CF obtained for the LSTM model, which was trained on raw data in the static case. Furthermore, it can be noticed that GBDT algorithms are the best-suited to dealing with attributes extracted from raw data. Similar results were achieved with all the considered subsets.

### 5.2. Unsupervised Methods

**Figure 9:**
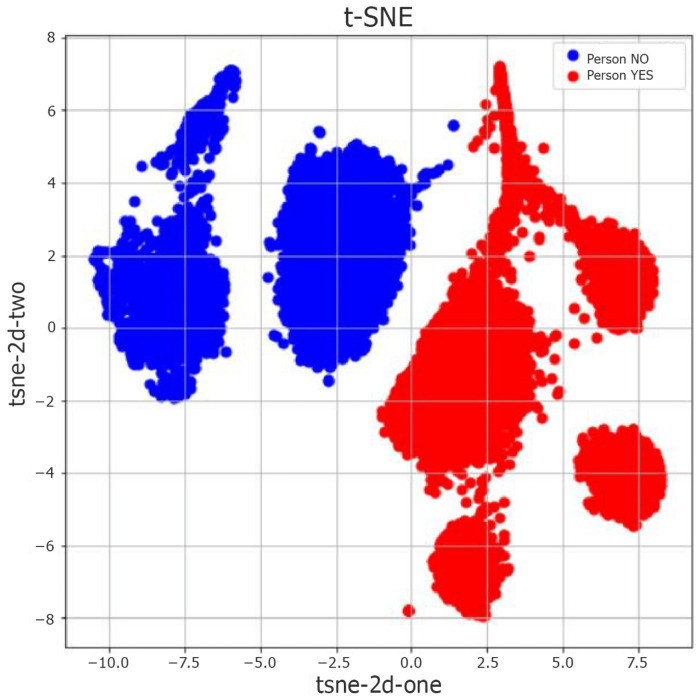
2D data projection with K-Means t-SNE model—all attributes, static case.

The performance metrics obtained for the unsupervised models are shown in Table 6 and Table 7 for the static and dynamic scenarios, respectively. Before applying the K-Means algorithm, we considered three different situations, in which the datasets were elaborated either without a dimensionality reduction step, by using PCA, or by developing t-SNE. We aimed to reduce the analysis to a two-dimensional problem and minimize the computational cost by preserving the most relevant information. The tables clarify that t-SNE is the best technique to work with K-Means, reaching an accuracy of about 74%. The two-dimensional projection of the statistical attributes dataset in the static case is illustrated in Figure 9; here, we highlight the two clusters found by the K-Means algorithm after the t-SNE dimensionality reduction.

**Figure 10:**
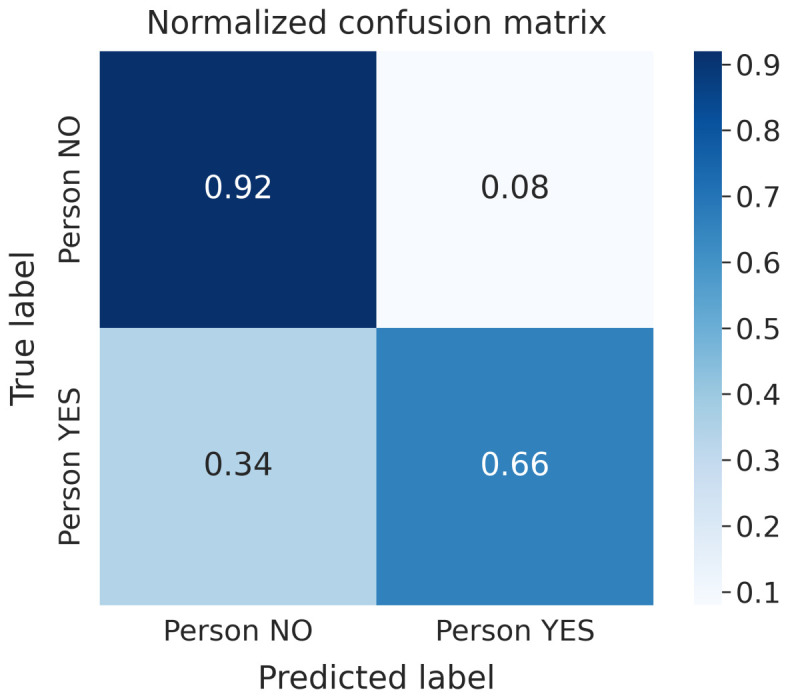
Normalized Confusion Matrix K-Means with t-SNE model—all attributes, static case.

The best models we obtained in both scenarios were trained on the horizontal transformed data and all the attributes extracted from the waveforms. These models are characterized by high Precision and low Recall for the “Person YES” class; for the “Person NO” class, the models show low Precision and high Recall. Such metrics prove that the system finds “Person YES” in just a few cases. However, in such cases, the predicted labels are correct compared to the training labels. On the other hand, the model returns many “Person NO”, even though such results are not always accurate. Moreover, our study highlights a small accuracy gap between the unsupervised models trained on static and dynamic datasets. Furthermore, clustering algorithms seem to work better with attributes extracted from raw data rather than with the waveforms themselves, as NNs and GBDTs do. The model we trained using all the attributes—whose normalized CF is shown in Figure 10—proved to be the best and most stable one.

### 5.3. Comparison with a Periodogram-Based TLC Algorithm

We compared the results described in Section 5.1 with a classical periodogram-based TLC approach (pseudocode in Algorithm 1, implemented in MatLab) to formally assess the benefits of ML techniques for general through-wall human detection. This algorithm studies the spectrum of the waveforms received by the UWB radar—which derive from the interaction between the transmitted train of Gaussian pulses and the surrounding environment (human target included)—to classify the presence of a person based on the identification of respiratory rate components. Normally, by breathing, a relaxed person can cause a displacement of the rib cage ranging from 0.1 mm to a few centimeters. Such movements occur with a frequency that is commonly between 0.2 and 0.7 Hz [36], which is possibly detectable by observing the harmonics of the received signal’s spectrum.

Starting from the raw matrices presented in Section 3, we followed three macro-steps:

- Static clutter removal (i.e., elimination of unwanted echoes, generally due to the reflection of electromagnetic energy on the natural and artificial elements surrounding the target);
- DC component removal;
- Signal spectrum computation via vertical FFT.

**Algorithm 1:** Periodogram-based TLC human detector
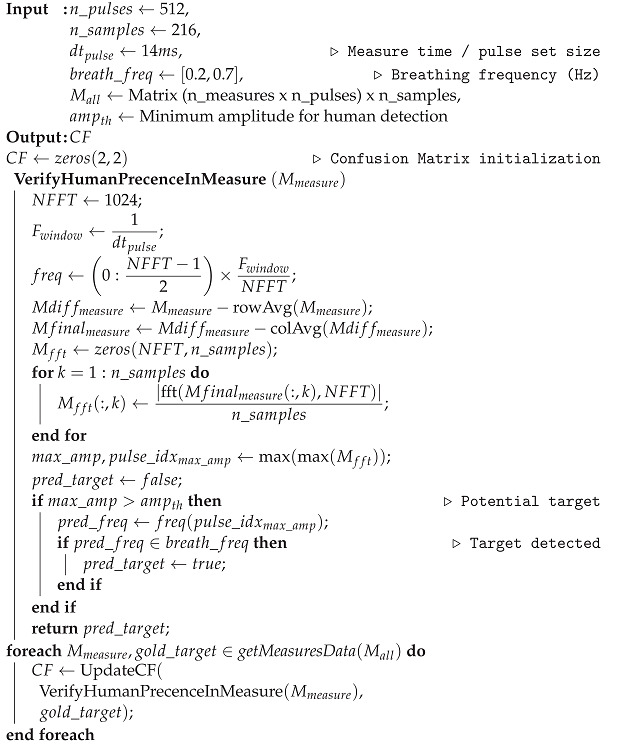


**Figure 11:**
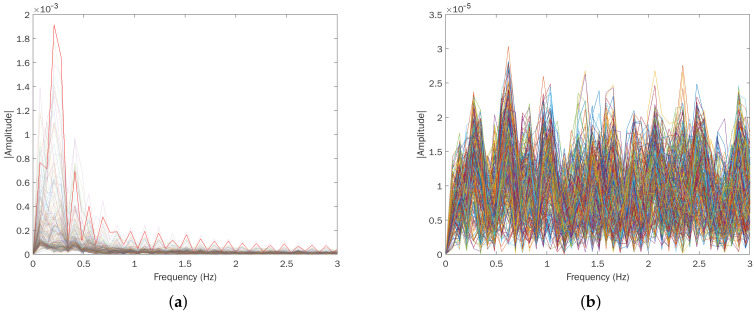
Simple spectrum comparison in LOS conditions between present and absent human target scenarios. (**a**) Signal spectrum with human target. The fundamental harmonic related to respiration is highlighted in red (≈13 breaths/min), while the others are at multiple frequencies with decreasing amplitudes. (**b**) Signal spectrum without human target.

Next, the harmonic with greater amplitude is searched in the spectrum; if it exceeds a certain amplitude threshold $amp_{th}$ and conforms to a Doppler compatible with the breath, the human target presence is deduced (Figure 11). Alternatively, the correspondence between the highest harmonic and a valid signal can be verified by calculating the difference between the predicted radar-target distance and the real one, using the well-known formula D=0.5×Δt×c, where Δt is the temporal distance and *c* is the light speed. However, we focused on the first case to avoid introducing and calibrating additional scenario-specific parameters besides $amp_{th}$, e.g., the delay introduced by the obstacle and the target for establishing Δt.

**Figure 12:**
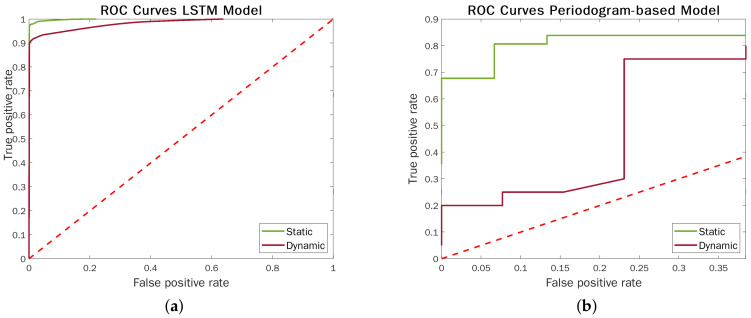
ROC curves LSTM vs. Periodogram-based TLC models in static and dynamic settings. The red dashed line indicates a random classifier. (**a**) ROC Curves LSTM model—raw data. Discriminating threshold = probability predicted by the model for the gold class. (**b**) ROC Curves Periodogram-based model—raw data. Discriminating threshold = minimum amplitude in the vertical FFT spectrum for human detection ($amp_{th}$).

We evaluated Algorithm 1 on a set of 600 $amp_{th}$ test values, ranging from 0 to 0.2. Figure 12 highlights the performance contrast between the LSTM models and the above-described approach in static and dynamic acquisitions. The ROC curves corroborate the strengths of ML techniques, which makes them significantly more effective and robust to radar semi-movements.

### 5.4. Generality of Neural Models and Transfer Learning

The experiments we illustrated above are all *in-domain* type, which means that the models are trained and tested over sub-sets belonging to the same dataset, namely, those belonging to the same scenario. Since our goal is to evaluate the generalization capacity of these models, we compare their accuracy by applying them to different scenarios concerning their training environment.

Adapting a pre-trained model for a target scenario different from the source one is known in ML literature as *transfer learning* (TL) [37]. TL has recently revolutionized many AI fields, especially natural language processing [38,39,40]. The generality of a model is an important aspect, particularly when new data signals for new scenarios cannot be labeled, such as in emergency situations or due to limits in time and cost sustainability. Potentially, the more general a model is, the more it could be online distributed without performing in loco new measurement campaigns.

In this work, the model generality has been evaluated by considering the static dataset as *source* because of the higher reliability of the acquired signals, and the dynamic one as *target*. This subsection reports two sets of experiments based on cross-domain with and without fine-tuning.

In the cross-domain experiments, we trained models on the static case data, while the test was carried out on dynamic datasets. We used this method to verify if the models obtained from one measurement campaign are also efficient for the other one.

**Figure 13:**
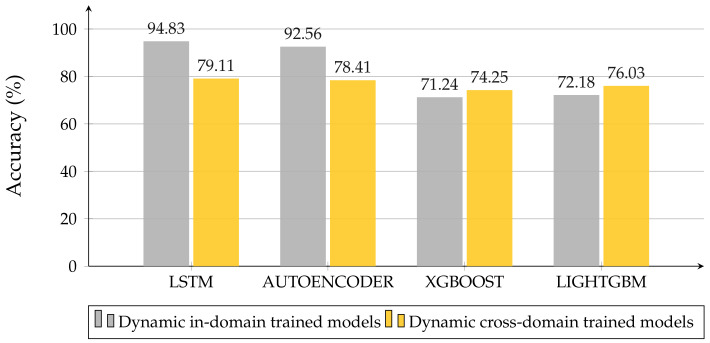
Comparison of best models trained on raw data—Cross-Domain experiment.

Figure 13 illustrates how the GBDT models, which were obtained for the static case and tested on dynamic data, present better performances than the dynamic ones, which were trained and tested in-domain. On the flip side, NN architectures are less adaptable, losing about 10–15% accuracy. Including the fine-tuning in the cross-domain experiments, we only worked with NN architectures. To take the lack of measurements into account, we trained the models obtained from the *Cross-Domain* evaluation again, by using small sub-sets of dynamic data. We evaluated three train-test divisions for the dynamic datasets: 10–90%, 20–80%, and 30–70%. In this way, not only do we want to verify if the models trained on static data suit the dynamic scenario, we also want to test if these models providee better results than the corresponding ones, trained in-domain (i.e., trained in the same condition without fine-tuning), even under lack-of-data conditions.

**Figure 14:**
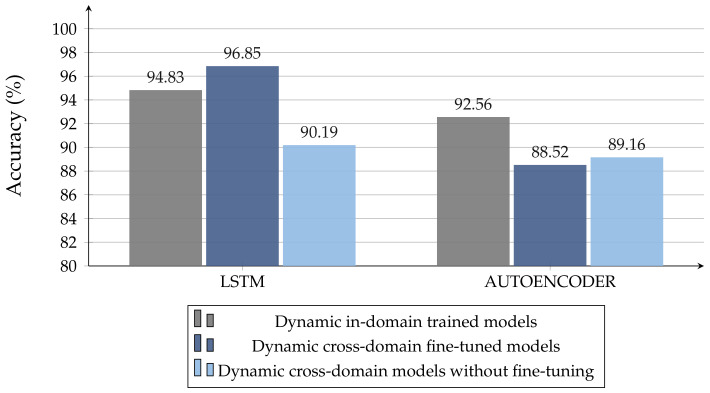
Comparison of best models trained on raw data (10%)—Cross-Domain with fine-tuning experiment.

It could be notied that NN models exhibit high generality, and they work very well even in with a lack of training data. From Figure 14, it is possible to observe how cross-domain LSTM models, trained with few measurements, work even better than the corresponding in-domain ones, trained on larger datasets. The latter consideration does not apply to AE architectures, for which the best model remains the one trained in-domain.

## 6. Discussion

Traditional TLC techniques based on detection theory achieve an excellent performance—better than any other approach—only if the observation model (signals, propagation, noise, clutter) is perfectly consistent with reality. However, if such a model is not known or only partially known, they may give poor results or require the calibration of unknown parameters, which risk being scenario-dependent (environment, wall material, radar positioning, etc.). Carrying out and repeating complex mathematical studies on waveforms and extensive parameter searches for each new usage scenario is unscalable and highly costly (considering both economics and time). Additionally, it is not possible for some real-world emergency scenarios, such as earthquake disasters, where the conformation of the rubble under which there could be trapped liives is completely arbitrary and not measurable a priori. ML approaches and recent breakthroughs in transfer learning can overcome these challenges, automatically learning and adapting model parameters to converge towards *a* maximum accuracy value, even in very complex dynamic conditions where the transponder is held in-hand (semi-movement).

To the best of our knowledge, this is the first work to compare such a variety of classifiers—supervised and unsupervised, symbolic and neural—on the task in question by considering: (i) various target orientations, obstacle materials, and radar-obstacle distances; (ii) raw data with and without Fast Fourier transformations and multiple sets of features; (iii) static and dynamic radar pulse signal acquisitions; (iv) generality towards unseen scenarios with a limited amount of training data. The same ML model can effectively deal with different environments and NLOS conditions, recognizing patterns that would be difficult to find otherwise. Our study highlights the effectiveness of deep learning models operating directly on time-domain waveforms as sequential inputs, where feature engineering—which is mostly helpful in unsupervised settings—is replaced by automatically learned dense and hidden representations. From our experiments, LSTM emerges as the most effective architecture, with an accuracy of 99.70% in static acquisitions and 94.83% in dynamic ones (96.85% through fine-tuning of static models, demonstrating the utility of overcoming the isolated learning paradigm in related tasks). The performances achieved by our deep neural models suggest their ability to recognize patterns related to breathing, generalizing to various obstacle types and data acquiring modalities. We hope that this paper will stimulate future research in the area, providing insights into the most effective configurations and encouraging the community to apply deep-learning advancements on UWB signals.

## 7. Conclusions

In this paper, several machine learning approaches for detecting human beings were analyzed using UWB signals. Starting from the data collected in an extensive measurement campaign in realistic environments, which considered different obstacles and body orientations, we extracted a knowledge model to classify the presence or absence of a person. We considered many supervised learning techniques and an unsupervised one, which does not require training data to be labeled. The former includes three GBDT algorithms and three NN architectures, whereas, in the latter, we used K-Means as a clustering method, combined with PCA and t-SNE as dimensionality reduction techniques. The goodness of fit of such models was evaluated with Accuracy, Precision, Recall, and F1-Measure.

Our classifiers outperformed similar works in the literature using raw data, without the need for large preprocessing phases or massive hyperparameter-tuning operations. Moreover, it was possible to obtain comparable or superior accuracy scores when generalizing the best models (e.g., LSTM) with transfer learning techniques. Our contributions could lead to software and hardware applications employing reliable human presence classification models even in limited-data settings, different or unseen scenarios, playing a huge role in life rescue, anti-terrorism, and other fields.

Future works may aim to achieve a similar performance by using simpler datasets with less significant attributes rather than attempting to improve classification model accuracy, which is already satisfactory. This result might be achieved using self-supervised Transformer models, as presented in [41]. As already deepened with different data modalities, we argue that combining symbolism and connectionism may be a promising avenue for the evolution of radar signal processing, emphasized by the urgent need for explainability [42,43].

## Figures and Tables

**Table 1:** Measurement setup.

Condition	Variants
Body orientations	no person
	person front faced (0 degrees)
	person in profile (90 degrees)
	person back faced (180 degrees)
Obstacle’s material	double-glazing *s* = 10 cm (only static case)
	wooden door *s* = 3 and 5 cm
	brickwall *s* = 15 cm
	glass window *s* = 2 cm
Radar-obstacle distance	*r* = 30 cm (only static case)
	*r* = 60 cm
	*r* = 90 cm
	*r* = 120 cm (only dynamic case)
Radar position	Static pose, 130 cm from the ground
	Dynamic pose

**Table 2:** Attributes calculated from each waveform.

Attribute’s Name	Meaning	Notation
Max	maximum value	$x_{Max}$
min	minimum value	$x_{min}$
Mean	mean value	$\bar{x}$
Std.deviation	distribution’s standard deviation	$\sigma_{X}$
Skewness	distribution’s skewness	$b_{1}$
Kurtosis	distribution’s kurtosis	$g_{2}$
Energy	signal’s Energy	*E*
Max/min	ration between max and min	$x_{Max}/x_{min}$
Max−min	difference between max and min	$x_{Max}-x_{min}$
SD/mean	std.deviation–mean ratio	$s/\bar{x}$
Max−minsqrd	max and min squared difference	${\left(x_{Max}-x_{min}\right)}^{2}$

**Table 3:** Datasets legend.

Dataset Name	Data Type
*raw data*	time-domain waveforms
*orz fft*	transformed raw data (horizontal FFT)
*ver fft*	transformed raw data (vertical FFT)
*all att*	all attributes from raw data
*4 att*	Max, Min, Mean, Std.Deviation
*E att*	Energy
*4+E att*	Max, Min, Mean, Std.Deviation, Energy
*SK att*	Skewness, Kurtosis

**Table 4:** Supervised methods—static case.

	Accuracy	Class	Precision	Recall	F1	Method
*raw data*	99.70%	YES	99.70%	97.82%	98.75%	LSTM
		NO	98.54%	99.15%	98.85%	
*orz fft*	97.10%	YES	97.10%	93.84%	95.44%	LSTM
		NO	97.98%	93.70%	95.79%	
*ver fft*	70.38%	YES	52.29%	83.43%	64.52%	AE
		NO	89.04%	64.16%	74.58%	
*all att*	89.86%	YES	90.30%	88.55%	88.19%	XGBoost
		NO	93.47%	92.33%	92.48%	
*4 att*	89.58%	YES	89.99%	88.15%	87.86%	XGBoost
		NO	93.10%	92.27%	92.28%	
*E att*	88.02%	YES	88.13%	86.67%	86.22%	XGBoost
		NO	92.29%	90.56%	91.02%	
*4+E att*	89.58%	YES	89.99%	88.15%	87.86%	XGBoost
		NO	93.10%	92.27%	92.28%	
*SK att*	87.21%	YES	87.07%	85.36%	85.18%	CatBoost
		NO	90.84%	90.96%	90.59%	

**Table 5:** Supervised methods—dynamic case.

	Accuracy	Class	Precision	Recall	F1	Method
*raw data*	94.83%	YES	92.01%	95.96%	93.94%	LSTM
		NO	97.01%	94.02%	95.49%	
*orz fft*	92.56%	YES	87.93%	95.26%	91.45%	AE
		NO	96.38%	90.62%	93.41%	
*ver fft*	83.87%	YES	77.65%	86.58%	81.87%	AE
		NO	89.37%	81.91%	85.48%	
*all att*	79.14%	YES	80.11%	78.65%	78.69%	CatBoost
		NO	80.50%	81.44%	80.51%	
*4 att*	79.30%	YES	80.77%	78.64%	78.81%	CatBoost
		NO	80.18%	82.40%	80.75%	
*E att*	78.10%	YES	79.39%	77.09%	77.38%	CatBoost
		NO	79.27%	82.83%	80.57%	
*4+E att*	79.22%	YES	80.62%	78.53%	78.72%	CatBoost
		NO	79.93%	82.43%	80.66%	
*SK att*	76.26%	YES	77.73%	75.34%	75.66%	CatBoost
		NO	76.32%	80.56%	77.90%	

**Table 6:** K-Means—static case.

	Accuracy	Class	Precision	Recall	F1	Method
*raw data*	52.31%	YES	66.61%	58.63%	62.36%	PCA
		NO	31.66%	40.86%	35.68%	
*orz fft*	74.61%	YES	95.86%	65.13%	77.57%	t-SNE
		NO	56.75%	94.19%	70.82%	
*ver fft*	52.79%	YES	68.73%	54.95%	61.07%	t-SNE
		NO	34.17%	48.32%	40.03%	
*all att*	74.37%	YES	94.54%	65.77%	77.57%	t-SNE
		NO	56.56%	92.17%	70.11%	
*4 att*	58.29%	YES	75.47%	56.46%	64.60%	t-SNE
		NO	41.09%	63.58%	49.92%	
*4+E att*	58.72%	YES	75.58%	57.23%	65.14%	t-SNE
		NO	41.16%	61.78%	49.41%	
*SK att*	75.64%	YES	94.81%	67.55%	78.89%	t-SNE
		NO	57.93%	92.36%	71.20%	

**Table 7:** K-Means—dynamic case.

	Accuracy	Class	Precision	Recall	F1	Method
*raw data*	52.02%	YES	60.98%	51.20%	55.66%	t-SNE
		NO	43.28%	53.20%	47.73%	
*orz fft*	72.82%	YES	82.72%	67.99%	74.63%	t-SNE
		NO	63.55%	79.72%	79.72%	
*ver fft*	54.89%	YES	63.01%	56.47%	59.56%	t-SNE
		NO	45.84%	52.64%	49.01%	
*all att*	73.55%	YES	82.06%	70.44%	75.81%	t-SNE
		NO	64.88%	78.00%	70.83%	
*4 att*	52.97%	YES	61.25%	54.55%	57.70%	t-SNE
		NO	43.86%	50.70%	47.04%	
*4+E att*	52.62%	YES	61.43%	52.28%	56.49%	t-SNE
		NO	43.80%	53.11%	48.01%	
*SK att*	65.23%	YES	73.34%	64.25%	68.49%	t-SNE
		NO	56.62%	66.64%	61.22%	

## Data Availability

Datasets are publicly available at https://github.com/disi-unibo-nlu/uwb-nlos-human-detection, accessed on 22 December 2021.

## References

[1] Salmi J., Molisch A.F. (2011). Propagation parameter estimation, modeling and measurements for ultrawideband MIMO radar. IEEE Trans. Antennas Propag..

[2] Ossberger G., Buchegger T., Schimback E., Stelzer A., Weigel R. Non-invasive respiratory movement detection and monitoring of hidden humans using ultra wideband pulse radar. Proceedings of the 2004 International Workshop on Ultra Wideband Systems Joint with Conference on Ultra Wideband Systems and Technologies, Joint UWBST IWUWBS 2004 (IEEE Cat. No.04EX812).

[3] Yarovoy A., Ligthart L., Matuzas J., Levitas B. (2006). UWB radar for human being detection. IEEE Aerosp. Electron. Syst. Mag..

[4] Zaikov E., Sachs J., Aftanas M., Rovnakova J. (2008). Detection of trapped people by UWB radar. Proceedings of the German Microwave Conference.

[5] Li J., Zeng Z., Sun J., Liu F. (2012). Through-wall detection of human being’s movement by UWB radar. IEEE Geosci. Remote Sens. Lett..

[6] Schleicher B., Nasr I., Trasser A., Schumacher H. (2013). IR-UWB radar demonstrator for ultra-fine movement detection and vital-sign monitoring. IEEE Trans. Microw. Theory Tech..

[7] Rittiplang A., Phasukkit P. (2020). 1-Tx/5-Rx Through-Wall UWB Switched-Antenna-Array Radar for Detecting Stationary Humans. Sensors.

[8] Li J., Liu L., Zeng Z., Liu F. (2013). Advanced signal processing for vital sign extraction with applications in UWB radar detection of trapped victims in complex environments. IEEE J. Sel. Top. Appl. Earth Obs. Remote Sens..

[9] Casadei V., Nanna N., Dardari D. (2011). Experimental study in breath detection and human target ranging in the presence of obstacles using ultra-wideband signals. Int. J. Ultra Wideband Commun. Syst..

[10] Kilic Y., Wymeersch H., Meijerink A., Bentum M.J., Scanlon W.G. (2013). Device-free person detection and ranging in UWB networks. IEEE J. Sel. Top. Signal Process..

[11] Patel A., Kosko B. (2009). Optimal noise benefits in Neyman-Pearson and inequality-constrained statistical signal detection. IEEE Trans. Signal Process..

[12] Gao Y., Li H., Himed B. (2018). Adaptive Subspace Tests for Multichannel Signal Detection in Auto-Regressive Disturbance. IEEE Trans. Signal Process..

[13] Hua X., Ono Y., Peng L., Cheng Y., Wang H. (2021). Target Detection Within Nonhomogeneous Clutter Via Total Bregman Divergence-Based Matrix Information Geometry Detectors. IEEE Trans. Signal Process..

[14] Rosli R.S., Habaebi M.H., Islam M.R. (2019). On the analysis of received signal strength indicator from ESP8266. Bull. Electr. Eng. Inform..

[15] Barral V., Escudero C.J., Suárez-Casal P., García-Naya J.A., Potortì F., Renaudin V., O’Keefe K., Palumbo F. (2019). Impact of NLOS identification on UWB-based localization systems. Proceedings of the Tenth International Conference on Indoor Positioning and Indoor Navigation—Work-in-Progress Papers (IPIN-WiP 2019) Co-Located with the Tenth International Conference on Indoor Positioning and Indoor Navigation (IPIN 2019).

[16] Yang D., Zhu Z., Zhang J., Liang B. (2021). The Overview of Human Localization and Vital Sign Signal Measurement Using Handheld IR-UWB Through-Wall Radar. Sensors.

[17] Khan U.M., Kabir Z., Hassan S.A., Ahmed S.H. A deep learning framework using passive WiFi sensing for respiration monitoring. Proceedings of the GLOBECOM 2017—2017 IEEE Global Communications Conference.

[18] Li Y., Wang W., Jiang Y. (2018). Through wall human detection under small samples based on deep learning algorithm. IEEE Access.

[19] Ding R., Li X., Nie L., Li J., Si X., Chu D., Liu G., Zhan D. (2019). Empirical Study and Improvement on Deep Transfer Learning for Human Activity Recognition. Sensors.

[20] Park J., Nam S., Choi H., Ko Y., Ko Y.B. (2020). Improving deep learning-based UWB LOS/NLOS identification with transfer learning: An empirical approach. Electronics.

[21] Moro G., Pasolini R., Dardari D. LOS/NLOS Wireless Channel Identification based on Data Mining of UWB Signals. Proceedings of the 8th International Conference on Data Science, Technology and Applications (DATA 2019).

[22] Domeniconi G., Semertzidis K., López V., Daly E.M., Kotoulas S., Moro G. (2016). A Novel Method for Unsupervised and Supervised Conversational Message Thread Detection. DATA 2016—Proceedings of the 5th International Conference on Data Science, Technology and Applications.

[23] Domeniconi G., Moro G., Pagliarani A., Pasini K., Pasolini R. (2016). Job Recommendation from Semantic Similarity of LinkedIn Users’ Skills. Proceedings of the 5th International Conference on Pattern Recognition Applications and Methods, ICPRAM 2016.

[24] Chen T., Guestrin C. Xgboost: A scalable tree boosting system. Proceedings of the 22nd ACM SIGKDD International Conference on Knowledge Discovery and Data Mining.

[25] Ke G., Meng Q., Finley T., Wang T., Chen W., Ma W., Ye Q., Liu T.Y. Lightgbm: A highly efficient gradient boosting decision tree. Proceedings of the Advances in Neural Information Processing Systems.

[26] Prokhorenkova L., Gusev G., Vorobev A., Dorogush A.V., Gulin A. CatBoost: Unbiased boosting with categorical features. Proceedings of the Advances in Neural Information Processing Systems.

[27] Schmidhuber J. (2015). Deep learning in neural networks: An overview. Neural Netw..

[28] Hochreiter S., Schmidhuber J. (1997). Long short-term memory. Neural Comput..

[29] Williams R.J., Zipser D. (1989). A learning algorithm for continually running fully recurrent neural networks. Neural Comput..

[30] Fabbri M., Moro G. (2018). Dow Jones Trading with Deep Learning: The Unreasonable Effectiveness of Recurrent Neural Networks.

[31] Lu X., Tsao Y., Matsuda S., Hori C. Speech enhancement based on deep denoising autoencoder. Proceedings of the Interspeech.

[32] Kieu T., Yang B., Guo C., Jensen C.S. Outlier Detection for Time Series with Recurrent Autoencoder Ensembles. Proceedings of the IJCAI.

[33] Géron A. (2019). Hands-on Machine Learning with Scikit-Learn, Keras, and TensorFlow: Concepts, Tools, and Techniques to Build Intelligent Systems.

[34] Abdi H., Williams L.J. (2010). Principal component analysis. Wiley Interdiscip. Rev. Comput. Stat..

[35] Maaten L.v.d., Hinton G. (2008). Visualizing data using t-SNE. J. Mach. Learn. Res..

[36] Lazaro A., Girbau D., Villarino R. (2010). Analysis of vital signs monitoring using an IR-UWB radar. Prog. Electromagn. Res..

[37] Pan S.J., Yang Q. (2009). A survey on transfer learning. IEEE Trans. Knowl. Data Eng..

[38] Domeniconi G., Moro G., Pasolini R., Sartori C. (2014). Iterative Refining of Category Profiles for Nearest Centroid Cross-Domain Text Classification. Proceedings of the 6th International Joint Conference on Knowledge Discovery (IC3K 2014).

[39] Domeniconi G., Moro G., Pagliarani A., Pasolini R. (2017). On Deep Learning in Cross-Domain Sentiment Classification. Proceedings of the 9th International Joint Conference on Knowledge DiscoveryIC3K 2017.

[40] Moro G., Pagliarani A., Pasolini R., Sartori C. (2018). Cross-domain & In-domain Sentiment Analysis with Memory-based Deep Neural Networks. Proceedings of the IC3K 2018.

[41] Mousavi S.M., Ellsworth W.L., Zhu W., Chuang L.Y., Beroza G.C. (2020). Earthquake transformer—An attentive deep-learning model for simultaneous earthquake detection and phase picking. Nat. Commun..

[42] Frisoni G., Moro G., Carbonaro A. (2021). A Survey on Event Extraction for Natural Language Understanding: Riding the Biomedical Literature Wave. IEEE Access.

[43] Lang P., Fu X., Martorella M., Dong J., Qin R., Meng X., Xie M. (2020). A Comprehensive Survey of Machine Learning Applied to Radar Signal Processing. arXiv.

